# Dosimetric analysis and comparison of IMRT and HDR brachytherapy in treatment of localized prostate cancer

**DOI:** 10.4103/0971-6203.62201

**Published:** 2010

**Authors:** V. Murali, P. G. G. Kurup, P. Mahadev, S. Mahalakshmi

**Affiliations:** Department of Radiation Oncology, Apollo Speciality Hospital, 320, Anna Salai, Chennai-600 035, India

**Keywords:** Brachytherapy, conformity, intensity modulated radiotherapy, prostate

## Abstract

Radical radiotherapy is one of the options for the management of prostate cancer. In external beam therapy, 3D conformal radiotherapy (3DCRT) and intensity modulated radiotherapy (IMRT) are the options for delivery of increased radiation dose, as vital organs are very close to the prostate and a higher dose to these structures leads to an increased toxicity. In brachytherapy, low dose rate brachytherapy with permanent implant of radioactive seeds and high dose rate brachytherapy (HDR) with remote after loaders are available. A dosimetric analysis has been made on IMRT and HDR brachytherapy plans. Ten cases from each IMRT and HDR brachytherapy have been taken for the study. The analysis includes comparison of conformity and homogeneity indices, D100, D95, D90, D80, D50, D10 and D5 of the target. For the organs at risk (OAR), namely rectum and bladder, V100, V90 and V50 are compared. In HDR brachytherapy, the doses to 1 cc and 0.1 cc of urethra have also been studied. Since a very high dose surrounds the source, the 300% dose volumes in the target and within the catheters are also studied in two plans, to estimate the actual volume of target receiving dose over 300%. This study shows that the prescribed dose covers 93 and 92% of the target volume in IMRT and HDR brachytherapy respectively. HDR brachytherapy delivers a much lesser dose to OAR, compared to the IMRT. For rectum, the V50 in IMRT is 34.0cc whilst it is 7.5cc in HDR brachytherapy. With the graphic optimization tool in HDR brachytherapy planning, the dose to urethra could be kept within 120% of the target dose. Hence it is concluded that HDR brachytherapy may be the choice of treatment for cancer of prostate in the early stage.

## Introduction

External beam radiotherapy (EBRT) for prostate cancer, using conformal technique, was practiced over many decades. According to Brenner and Hall,[1] for prostate cancer, the tumor and the surrounding late responding normal tissues are likely to have similar α/β values and thus similar sensitivities to changes in fractionation. The result is that healthy tissues can only be spared by the reduction of dose. On the other hand, increasing the dose to the tumor improves local control and survival. The only way to increase the dose to the tumor, without delivering more doses to the healthy tissues, is to make the dose distribution conformal. After the advent of IMRT, EBRT has become the choice of treatment for cancer of prostate. The IMRT has its own advantages; one of which is the treatment is non-invasive. The patient doesn't need hospitalization during the course of treatment. The dose distribution could be optimized to cover the target volume and minimize the dose to rectum. Hence the target dose could be escalated.

Brachytherapy to prostate became available as early as 1911[2] using Radium needles. Transrectal ultrasound based (TRUS) brachytherapy using Iodine-125 (125 I) seeds came into practice in the 1980s.[2] After the invention of high dose rate (HDR) remote after-loading machines, in the early 80's, removable implants were done.[3] There are advantages with removable implants with HDR machines, like no radiation exposure to the staff involved and short course of treatment for the patient. The catheters are implanted under TRUS guidance, using template. The source positions (dwell positions) and dwell time could be optimized for better target coverage while minimizing the dose to urethra and rectum. The catheters can be positioned outside the capsule to treat the extra capsular disease also. In EBRT, the patient is immobilized for treatment set-up reproducibility and daily setup verification is done by matching either the portal images with the DRR generated from the planning system, or the cone beam CT images (CBCT) with the CT images used for planning. The intra-fraction movement of the prostate could, however, be hardly controlled. With the removable implants, this issue doesn't arise at all. The prostate is held in position by the catheters and the catheters are held in position by the template, which is sutured to the perineum.

The objective of this study is to make a dosimetric comparison based on physical dose distribution, between IMRT and HDR brachytherapy (given as mono therapy), for the treatment of cancer of the prostate

## Materials and Methods

### Intensity modulated radiation therapy

**Figure 1:**
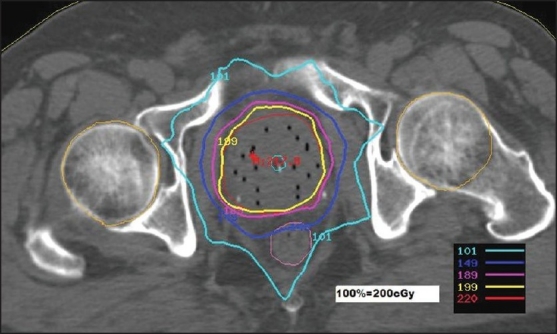
Dose distribution in IMRT plan- absolute dose per fraction

Ten treatment plans from each modality of treatment are selected for comparison. The treatment plans for IMRT were based on CT images acquired at 3 mm intervals. The OAR considered are, bladder, rectum and femoral heads. All the IMRT plans are done with seven coplanar beams of 6MV x-rays using the Plato Sun Rise Planning System (Nucletron, The Netherlands). The beams are placed almost at equal intervals of the gantry angles. The system uses gradient search method for the optimization, based on the user defined dose constraints and weights. The IMRT plans are MLC based, with step and shoot method. The width of the leaf at isocenter is 1 cm and the beam-let size is 1cm × 1cm. The target is the planning target volume (PTV), which includes the prostate and a 5mm margin around it. The margin is reduced to 3mm near the bladder and rectum. A dose of 76 Gy in 38 fractions is prescribed to the target. The total number of segments in a plan is approximately 70. The dose distribution of a typical plan is shown in Figure 1.

### Remote after-loading brachytherapy

A TRUS is done two days prior to the implant to map the prostate and to estimate the approximate number of catheters required. Nucletron prostate template is used for implant. The template has pre-drilled holes at 0.5 cm spacing with a square grid arrangement. Transperineal insertion of the catheters is done under TRUS. Post implant treatment plans are done on CT images acquired at 3mm intervals. The CT images are acquired roughly an hour after implantation is over. Rectum, bladder and urethra are taken as the OAR. While contouring the bladder and rectum, the outer most mucosa is contoured. For the urethra the outer surface of the Foley's is contoured. Plans are done with Iridium-192 (192 Ir) stepping source, using Plato Sun Rise Planning System. The step size for the dwell position used is 2.5mm. Dose points are defined on the periphery of the target volume at 8mm interval and optimization is made on these target dose points with a gradient factor of 0.5. A dose of 30 Gy in three fractions is prescribed to the target. One fraction is given on the day of the implant and the remaining two fractions on the following day.

**Figure 2:**
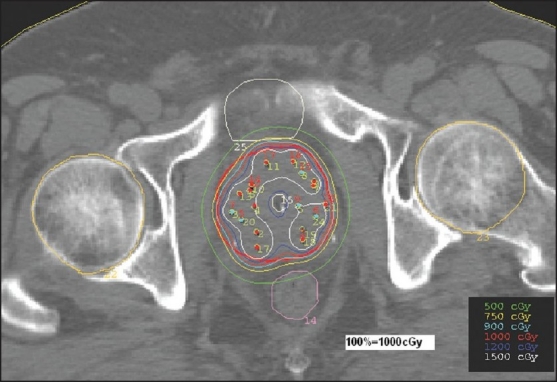
Dose distribution in HDR brachytherapy plan- absolute dose per fraction

Optimization in the brachytherapy module is different from the one in the inverse planning of external beam therapy module. The optimization plays with the dwell times and dwell positions. Only the dose points described on the target volume are considered for the optimization. No consideration is given for the hot spot inside the target. The objective is to achieve maximum coverage of target to the prescribed dose. No heterogeneity correction is applied and calculations are done for homogeneous tissue medium. Studies have been reported on the use of inverse planning with simulated annealing (IPSA) for HDR brachytherapy.[4,5] After the dose point optimization, graphic optimization is performed by dragging the isodose lines in such a way that the 125% and 150% isodose lines are mostly towards the boundary of the capsule and the dose to urethra doesn't exceed 125%. Sometimes ‘manual dwell weights’ for individual source position are adjusted to obtain the required dose distributions and make sure that dwell time is not less than one second. This is especially done when the source activity is between 9 and 10 Ci. A typical dose distribution is shown in Figure 2.

### Comparison of dosimetry

For the comparison of dosimetry or dose distribution of plans, the following criteria are considered:

1. Conformity of the prescribed dose to the target
2. Homogeneity of dose within the target volume
3. Dose to the organs at risk

**Figure 3:**
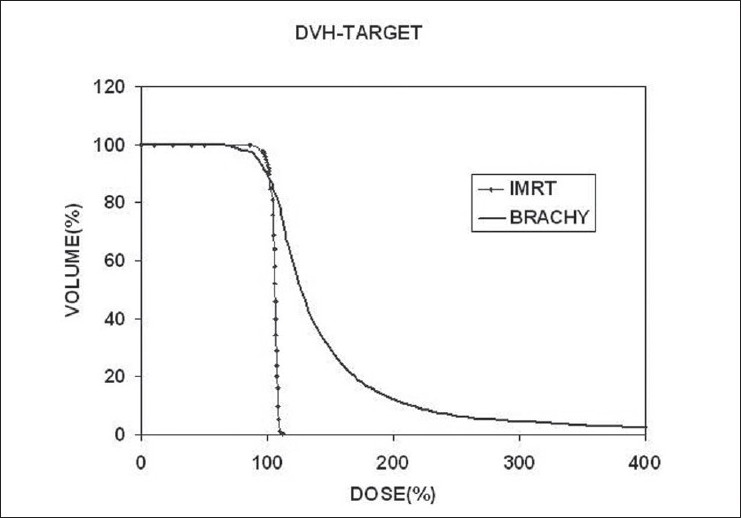
Comparison of DVHs of PTV

For target dose comparison, the best quantitative evaluation can be done with the use of dose volume histogram (DVH). The DVH is a good tool to evaluate a complex dose distribution;[6] but unfortunately it does not provide information with regard to the location of the global maximum dose within the target volume. In our study, we compared D_100_, D_98_, D_90_, D_80_, D_50_, D_10_ and D_5_ of the target [Tables 1 and 2]. Since the absolute dose prescribed for the IMRT and brachytherapy are different, the prescription dose (reference dose) to target volume is normalized to 100% and the relative dose values are compared. Figure 3 shows the comparative DVH of the target. The target dose uniformity and conformity are also evaluated and compared. The conformity index (CI), as defined in ICRU[7] is,${\text{CI}}_{\left(\text{ref}\right)}=\frac{\text{Volume of PTV covered by the reference dose}}{\text{Volume of PTV}}$

**Table 1:** Dose to PTV from IMRT

*Study No.*	*PTV (cc)*	*% Dose to PTV*							*Vol(%) of 100% dose*
			
		*D_100_*	*D_98_*	*D_90_*	*D_80_*	*D_50_*	*D_10_*	*D_5_*	*V_100_*
1	78.4	79.4	98.6	100.7	102.7	105.8	109.6	110.6	91.6
2	104.4	86.5	94.6	104.2	106.2	109.7	114.0	116.0	96.0
3	82.1	83.3	90.0	100.8	103.3	107.1	110.2	110.8	92.1
4	72.3	79..9	96.2	105.1	106.9	110.0	115.0	116.0	96.5
5	77.6	87.8	94.3	104.1	105.9	108.8	112.2	113.7	87.8
6	90.9	86.9	91.0	100.8	103.7	107.6	110.6	111.2	92.4
7	86.8	80.8	89.5	104.0	106.0	107.9	112.2	113.1	94.0
8	112.3	90.3	93.6	100.5	103.6	107.7	111.6	112.5	91.1
9	113.2	78.2	93.9	103.4	104.8	107.8	111.5	112.0	95.0
10	89.3	71.4	90.9	101.2	102.7	104.2	106.0	107.1	93.3
Mean	90.7	82.6	93.3	102.5	104.6	107.7	111.3	112.3	93.0
P-Value		0.0199	0.0003	0.0001	0.0001	0.0001	0.0001	0.0001	0.0001

**Table 2:** Dose to PTV from HDR brachytherapy

*Study No.*	*PTV (cc)*	*% Dose to PTV*							*Vol.(%) of 100% dose*
			
		*D_100_*	*D_98_*	*D_90_*	*D_80_*	*D_50_*	*D_10_*	*D_5_*	*(V_100_)*
1	62.0	71.9	82.3	97.1	108.2	127.0	205.5	274.0	88.5
2	34.2	76.6	94.6	107.8	115.7	141.9	256.3	335.0	96.1
3	77.4	70.1	86.2	102.4	110.0	126.0	201.9	257.0	91.9
4	96.8	84.0	97.1	108.1	114.3	134.0	234.0	305.9	96.9
5	39.4	79.8	94.3	102.0	110.0	129.0	255.0	349.0	92.7
6	67.8	69.3	91.0	98.0	108.0	137.0	243.0	349.0	88.2
7	81.4	79.2	92.5	103.8	114.5	129.5	213.4	273.2	93.5
8	52.8	69.7	93.6	100.8	107.6	127.4	237.2	330.8	90.9
9	110.7	75.3	91.1	104.4	111.4	127.4	195.8	244.3	93.7
10	89.3	68.5	90.9	99.2	107.7	126.9	213.0	349.0	89.4
Mean	71.2	74.4	91.4	102.4	110.7	130.6	225.5	306.7	92.2
P-value		0.0285	0.0040	0.0009	0.0002	0.0018	0.0721	0.1351	0.0004

CI= 1.00 is for an ideal case.

The conformity index is a scalar quantity and by itself cannot represent the true conformity.[8] Several indices have been proposed for the evaluation of dose distribution of brachytherapy implants.[9,10] Meertens *et al*,[10] have mentioned that the clinical significance and the correlations of these indices were not yet clear. Since this is a comparative study, it is decided not to use two different methods to evaluate the homogeneity of IMRT and brachytherapy plans. The Homogeneity Index (HI)[6] is calculated as follows

HI=D5−D95DP

where D_p_ is the prescribed dose to the target. The conformity and homogeneity indices are shown in the Tables 3 and 4 respectively.

**Table 3:** Comparison of conformity index

*Study No.*	*IMRT*			*Study No.*	*Brachytherapy*		
				
	*PTV (cc)*	*Vol.covered by ref.dose(cc)*	*(CI)*		*PTV (cc)*	*Vol.covered by ref.dose(cc)*	*(CI)*
1	78.4	71.8	0.916	1	62.0	54.9	0.885
2	104.4	100.2	0.960	2	34.2	32.9	0.962
3	82.1	75.6	0.921	3	77.4	71.1	0.919
4	72.3	69.8	0.965	4	96.8	93.8	0.969
5	77.6	68.1	0.878	5	39.4	36.5	0.927
6	90.9	84.0	0.924	6	67.8	59.8	0.882
7	86.8	81.6	0.940	7	81.4	76.1	0.935
8	112.3	102.3	0.911	8	52.8	48.0	0.909
9	113.2	107.5	0.950	9	110.7	103.7	0.937
10	89.3	83.3	0.933	10	89.3	80.2	0.894
Mean	90.7	84.4	0.930	Mean	71.2	65.7	0.922
P-value			0.0001	P-value			0.0005

**Table 4:** Comparison of homogeneity index

*Study No.*	*IMRT*			*Study No.*	*Brachytherapy*		
			
	*D_5_(%)*	*D_95_(%)*	*HI*		*D_5_(%)*	*D_95_(%)*	*HI*
1	110.6	99.3	0.123	1	274.0	89.7	1.84
2	116.0	102.2	0.138	2	335.1	102.0	2.33
3	110.8	98.6	0.122	3	257.0	94.6	1.62
4	116.0	103.6	0.124	4	305.9	102.8	2.03
5	113.7	102.1	0.116	5	349.0	96.1	2.52
6	111.2	97.5	0.137	6	349.0	90.3	2.58
7	113.1	101.7	0.114	7	273.2	98.1	1.75
8	112.5	94.6	0.179	8	330.8	94.3	2.36
9	112.0	101.7	0.103	9	244.3	97.9	1.46
10	107.1	98.3	0.088	10	284.2	92.5	1.91
Mean	112.3	100.0	0.124	Mean	300.3	95.8	2.04
P-value			0.2302	P-value			0.2142

**Figure 4:**
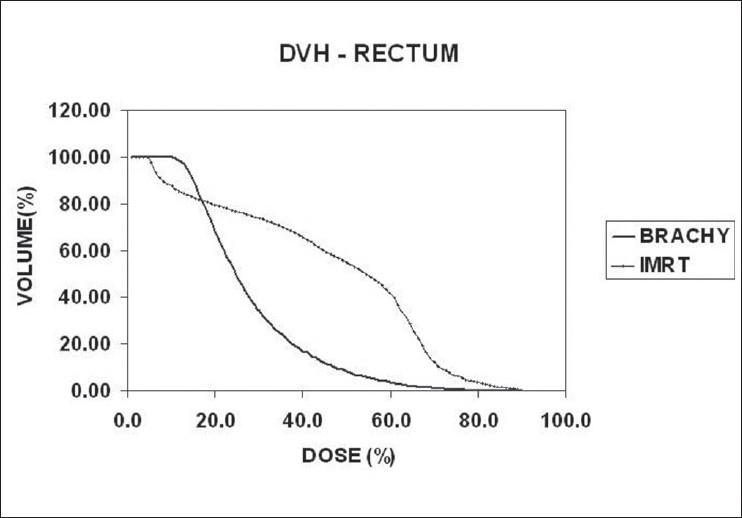
Comparison of DVHs of rectum

**Figure 5:**
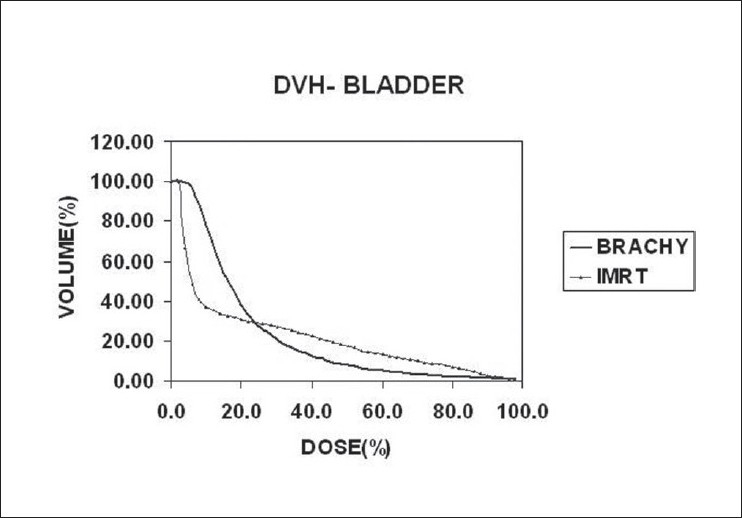
Comparison of DVHs of bladder

In radiation therapy, we are not only concerned with the dose received by the specified target volume, but also that received by the organs around the target, which are at risk. Although DVH is a useful tool to represent the statistics of dose distribution, its applicability to hollow organs such as esophagus and rectum remains unresolved.[11–15] Moreover, in the present study, the brachytherapy calculation algorithm considers the hollow structures also as unit density tissue medium. Some authors have compared the DVH, the dose-surface histogram (DSH) and dose-wall histogram (DWH) for cylindrical and spherical models of rectum and bladder respectively.[11,12,15,16] In this study, the actual volumes of rectum and bladder receiving the 100%(V_100_), 90%(V_90_) and 50%(V_50_) of the target dose are compared [Tables 5 and 6]. The DVHs of rectum and bladder are illustrated in Figures 4 and 5. The dose to urethra [Table 7] is studied in brachytherapy for two reasons. One, the dose per fraction is 10Gy and the other one is, in the immediate vicinity of sources, the dose is very high, which is the very nature of brachytherapy. In IMRT, as the prescribed dose per fraction is only 2Gy to the target and the maximum dose in the target doesn't exceed 115% [Table 1], the urethral dose is not of great concern.

**Table 5:** Comparison of V_100_, V_90_ and V_50_ of rectum

*Study No.*	*IMRT Volume in cc*			*Study No.*	*Brachytherapy Volume in cc*		
			
	*V_100_*	*V_90_*	*V_50_*		*V_100_*	*V_90_*	*V_50_*	
1	0.0	1.5	22.0	1	0.3	0.8	7.0
2	0.0	4.0	34.7	2	0.0	0.2	4.7
3	0.0	2.6	45.2	3	0.0	0.1	4.6
4	0.8	6.3	33.4	4	0.3	1.2	12.9
5	2.6	14.3	47.2	5	0.0	0.0	4.0
6	0.0	0.0	35.4	6	0.0	0.0	6.2
7	0.0	1.7	31.3	7	0.2	0.8	12.6
8	0.0	0.8	32.5	8	0.1	0.5	7.4
9	0.9	9.8	36.6	9	0.2	0.6	11.3
10	0.0	1.0	25.5	10	0.0	0.0	4.1
Mean	0.4	4.2	34.4	Mean	0.1	0.4	7.5
P-value	0.4708	0.4446	0.2489	P-value	0.3648	0.4971	0.3658

**Table 6:** Comparison of V_100_, V_90_ and V_50_ of bladder

*Study No.*	*IMRT Volume in cc*			*Study No.*	*Brachytherapy Volume in cc*		
			
	*V_100_*	*V_90_*	*V_50_*		*V_100_*	*V_90_*	*V_50_*	
1	0.0	6.2	48.8	1	2.5	3.4	15.1
2	0.0	7.5	131.9	2	0.0	0.0	2.2
3	1.6	10.6	62.2	3	0.2	0.3	4.2
4	2.8	15.1	73.4	4	2.0	3.0	12.9
5	9.8	19.5	88.1	5	0.4	0.8	7.6
6	0.0	12.4	114.1	6	2.3	3.2	15.5
7	2.6	11.6	71.0	7	0.8	1.6	12.0
8	0.0	5.2	45.0	8	2.4	3.6	16.7
9	0.0	3.6	61.9	9	2.6	3.6	18.4
10	0.0	6.3	38.2	10	2.0	3.4	18.7
Mean	1.7	9.8	73.5	Mean	1.5	2.5	12.3
P-value	0.4655	0.3813	0.3530	P-value	0.4115	0.2497	0.3852

**Table 7:** % Dose to Urethra in HDR Brachytherapy

*Study No.*	*1 cc*	*0.1cc*
1	111.7	124.7
2	95.8	116.7
3	108.9	117.1
4	111.1	119.4
5	103.9	117.8
6	111.2	123.8
7	113.2	119.6
8	107.2	122.8
9	112.9	118.9
10	109.8	120.0
Mean	108.6	120.1
P-value	0.0049	0.0001

### Radiobiological aspect

Even though the radiobiological aspect is beyond the scope of this article, the biological equivalence between IMRT and brachytherapy is found out purely for academic interest. The biologically effective dose (BED) is calculated for both the IMRT and brachytherapy using the equation, suggested by Fowler[17] ${\text{BED}}_{\text{fr}}=\text{N d}\left\{1+\text{d/}\left(\alpha /\beta \right)\right\}$

where N – the no. of fractions, d – the dose per fraction in Gy and α/β the tissue specific parameter. α/β value isassumed to be 3.1Gy for the tumor.[18] The above equation does not take into account the tumor cell proliferation factor. The BED values calculated for IMRT (76 Gy =2 Gy × 38#) and brachytherapy (30 Gy=10.0 Gy × 3 #) are 125.0Gy and 126.7Gy respectively. This shows that the dose regimens followed are radio-biologically equivalent.

## Results and Discussion

The *P*-values mentioned in the tables are estimated using student t-score method. From Tables 1 and 2 it is seen that the target volume ranges from 70 -113 cc with a mean volume of 91 cc in IMRT. In brachytherapy the target volume ranges from 34-110cc with a mean of 71 cc. It is seen that the mean value of D_90_ is almost the same in both IMRT and brachytherapy. In brachytherapy the D_100_ and D_98_ are slightly lower compared to IMRT. One of the reasons for this is the inability to insert catheters especially on the lateral and anterior aspects due to narrowing of the pubic arch. As the catheters are inserted through the template, there is very limited scope to manipulate the catheters to reach the target.

In this study, the mean value of D_5_ in IMRT is 112%, whereas in brachytherapy it is as high as 300%. A comparative DVH of the target is shown in Figure 3. In this analysis we have considered D_5_ and not D_2_ for the maximum dose within the target; for two reasons - one, in brachytherapy, total volume of the catheters constitute about 4-5% of the volume of the target. Of course, this value changes with the number of catheters used and the volume of the prostate. The other, within the catheters the dose would be immensely high to be considered as high dose within the tissue. To establish this fact, the 300% dose within the catheters, are calculated for study nos. 7 and 10, who were implanted with 17 and 18 catheters, respectively. Those parts of the catheters, which are in the target volume alone, are marked on CT images and considered for the analysis. In study no.7, volumes of the 300% dose within the target and within the catheters are 3.12 cc and 1.53 cc respectively. Hence the net volume of target tissue is 1.59 cc. This is only 1.95% for the target volume of 81.4cc, whereas the volume of catheters that receive 300% dose is 1.87%. For the study no.10, it is found that the volume of the target tissue is 2.84% and that of the catheters is 1.63% of the target volume. In both these cases, the volume of the catheters receiving 300% dose is close to 2% of the target volume and hence considering D_2_ as the maximum target dose may not be correct.

The following formula could be used to find the actual volume of the target that receives the high doses in the region of 300 to 350%, which are formed in and around the catheters and especially when the catheters are very close.

VACT=VPTV−ΣCTH

V_ACT_ – Actual volume of target that receives a particular % dose

V_PTV_ – Total volume of target that receives the particular % dose

∑_CTH-_ Total volume of catheters that receive the particular % dose

For illustration, the details are given for study no.10 only, in the Table 8. The study no.10 listed in Tables 1 and 2 underwent brachytherapy only. For the sake of comparison and analysis, the same CT images [Figures 1 and 2] with structure set are used to plan IMRT with the usual protocol. On comparing the DVHs of OAR [Figures 4 and 5], it is seen that the dose below 20%, is more in brachytherapy than in IMRT. The reason is very clear - that in brachytherapy optimization, the dose to OAR is not considered. In graphical optimization, only the high dose regions are adjusted.

**Table 8:** Dose Vs volume of PTV and catheters in HDR brachytherapy

*Dose (%)*	*Volume (cc)*		
	
	*PTV*	*Catheters*	*Actual*
	V_PTV_	∑_CTH_	V_ACT_
10	89.30	3.22	86.08
25	89.30	3.22	86.08
50	89.30	3.22	86.08
75	88.84	3.22	85.62
100	80.24	3.19	77.05
150	26.83	2.58	24.25
200	11.31	2.06	9.25
250	6.40	1.69	4.71
300	4.00	1.46	2.54
350	2.89	1.26	1.63
400	2.14	1.09	1.05
450	1.60	0.97	0.63

## Conclusions

1. The difference in target volume coverage between IMRT and brachytherapy plans is not very significant as optimization is done for the target volume in both the plans.
2. The mean conformity index 0.933 in IMRT is slightly better compared to the value of 0.922 in brachytherapy. Even though the dose distribution in the brachytherapy could be optimized and improved, it still largely depends on the implant
3. The homogeneity index is very good in IMRT. For the brachytherapy plans, probably new indices could be used to evaluate the homogeneity considering the very high dose within the target volume.

The temporary implant with HDR brachytherapy provides an acceptable dose distribution to the prostate in terms of coverage and a much-reduced dose to rectum and bladder, compared to the IMRT. The only concern is the homogeneity of dose within the target. By performing optimization in the HDR brachytherapy planning, the dose to urethra could also be kept within 120% of the target dose. From the dosimetry point of view, HDR brachytherapy would be a better choice for the treatment of early cancer of the prostate.
